# HOW TO GET AN NIA GRANT

**DOI:** 10.1093/geroni/igae098.0784

**Published:** 2024-12-31

**Authors:** Kenneth Santora

**Affiliations:** National Institute on Aging, Baltimore, Maryland, United States

## Abstract

Dr. Santora will provide an overview of the NIA application process, funding opportunities, and relevant policy changes for early-career researchers.

